# ThermoMutDB: a thermodynamic database for missense mutations

**DOI:** 10.1093/nar/gkaa925

**Published:** 2020-10-23

**Authors:** Joicymara S Xavier, Thanh-Binh Nguyen, Malancha Karmarkar, Stephanie Portelli, Pâmela M Rezende, João P L Velloso, David B Ascher, Douglas E V Pires

**Affiliations:** Institute of Agricultural Sciences, Universidade Federal dos Vales do Jequitinhonha e Mucuri; Instituto René Rachou, Fundação Oswaldo Cruz; Bio 21 Institute, University of Melbourne; Bio 21 Institute, University of Melbourne; Computational Biology and Clinical Informatics, Baker Heart and Diabetes Institute; Bio 21 Institute, University of Melbourne; Computational Biology and Clinical Informatics, Baker Heart and Diabetes Institute; Instituto René Rachou, Fundação Oswaldo Cruz; Instituto René Rachou, Fundação Oswaldo Cruz; Bio 21 Institute, University of Melbourne; Computational Biology and Clinical Informatics, Baker Heart and Diabetes Institute; Department of Biochemistry, University of Cambridge; Bio 21 Institute, University of Melbourne; Computational Biology and Clinical Informatics, Baker Heart and Diabetes Institute; School of Computing and Information Systems, University of Melbourne

## Abstract

Proteins are intricate, dynamic structures, and small changes in their amino acid sequences can lead to large effects on their folding, stability and dynamics. To facilitate the further development and evaluation of methods to predict these changes, we have developed ThermoMutDB, a manually curated database containing >14,669 experimental data of thermodynamic parameters for wild type and mutant proteins. This represents an increase of 83% in unique mutations over previous databases and includes thermodynamic information on 204 new proteins. During manual curation we have also corrected annotation errors in previously curated entries. Associated with each entry, we have included information on the unfolding Gibbs free energy and melting temperature change, and have associated entries with available experimental structural information. ThermoMutDB supports users to contribute to new data points and programmatic access to the database via a RESTful API. ThermoMutDB is freely available at: http://biosig.unimelb.edu.au/thermomutdb.

## Graphical Abstract

**Graphical Abstract:**
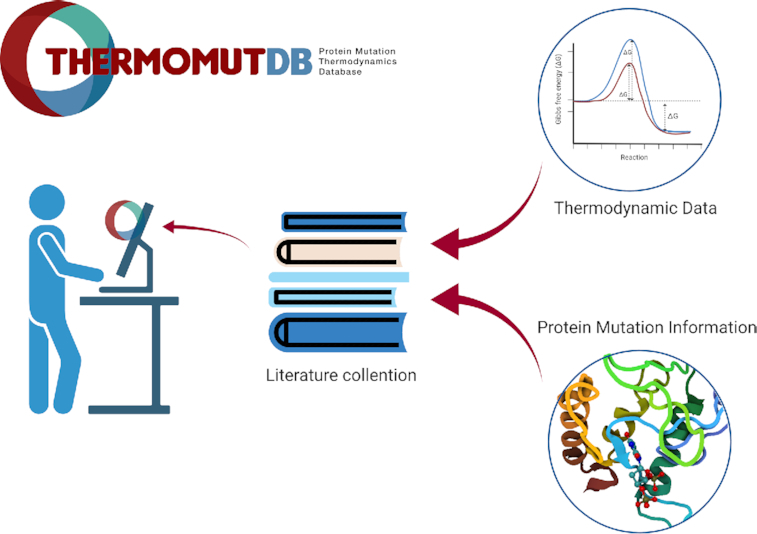
ThermoMutDB is an online resource associating effects of missense mutations on protein thermodynamics.

## INTRODUCTION

Protein thermodynamic stability is a fundamental property of proteins that significantly influences their structure, function, expression, and solubility. Changes in protein stability have been shown to be a main driving molecular mechanism of genetic diseases (1–8) and even drug resistance (9–18). Small changes in the protein sequence can have significant consequences on their intricate structures, reflected in changes in their stability and ability to correctly fold (19). This is often a significant consideration whenever considering a new mutation, whether in the context of protein engineering or variant characterisation (20,21).

The accurate prediction of the effects of mutations on protein stability remains a complex and challenging problem. The development of computational approaches to tackle this have required large mutational datasets, however in turn have been limited by the quantity and quality of data available.

One of the first databases to collect information on the effects of mutations on protein stability, ProTherm (22), led to the exploration and rapid development of new computational approaches (23–28). However, this database has not been updated for 7 years and many errors have been identified previously (29,30), limiting both previous methods and future developments.

**Figure 1.**
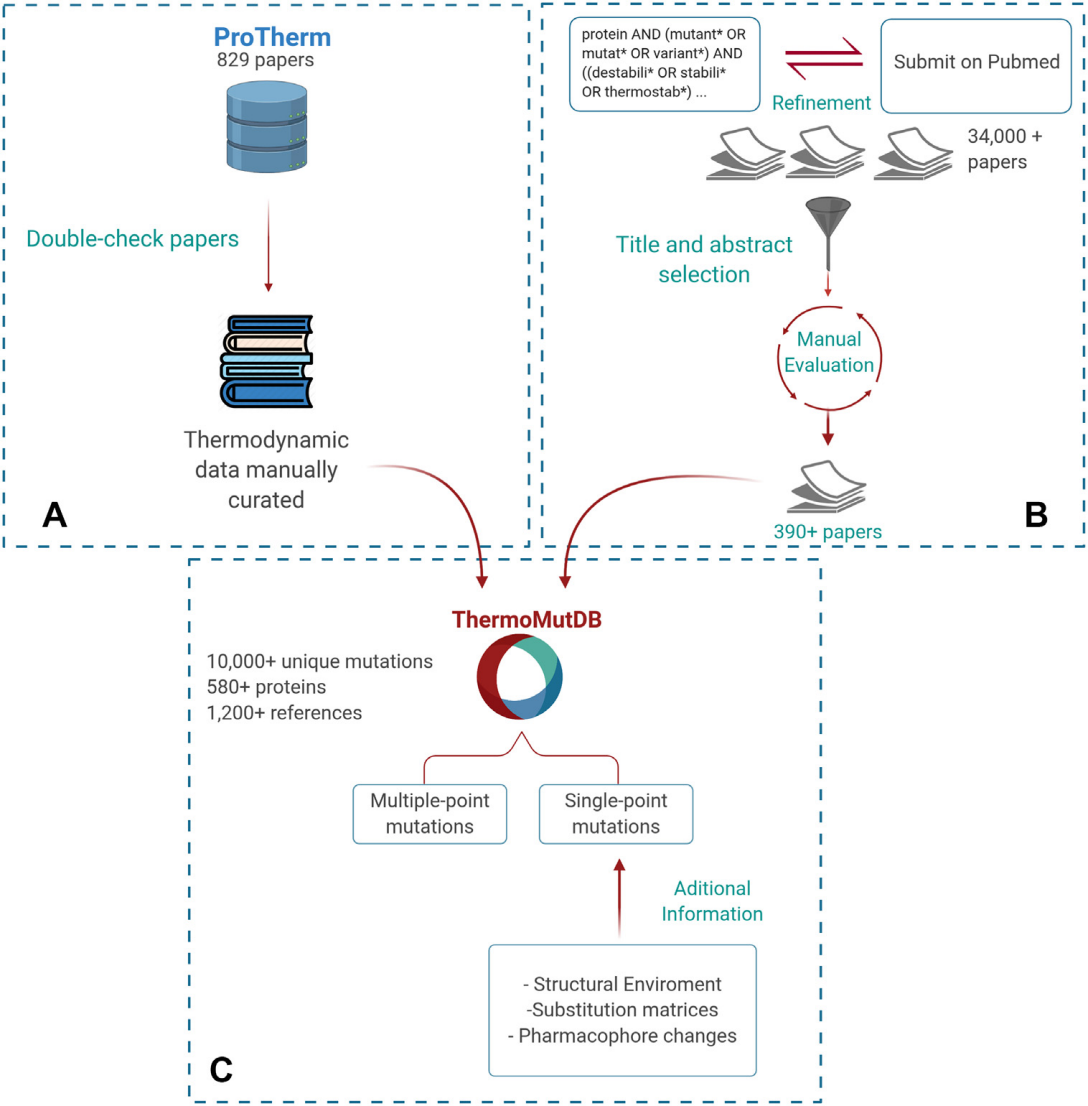
ThermoMutDB workflow for data acquisition and processing. The development workflow is divided into three steps: (**A**) verification of previously available mutation thermodynamics information (**B**) collection and manual curation of new data and (**C**) data aggregation and mutation annotation.

To overcome this, we have developed a new comprehensive and user-friendly resource for thermodynamic data from protein mutations, ThemoMutDB. Figure 1 depicts the database development workflow, which is divided into three main stages: (i) data acquisition and curation, (ii) mutation annotation and (iii) web-server development. By using a rigorous and careful data curation approach, ThemoMutDB represents a significant improvement in both the quantity and quality of data. This will not only enable the development of a new generation of methods but also an unbiased assessment of previously proposed ones.

## MATERIALS AND METHODS

### Data acquisition and curation

Data acquisition for ThermoMutDB was divided into two steps: manual checking of previously mined data available in other resources (Figure 1A) and manual literature curation of new thermodynamic data (Figure 1B). Within ThermoMutDB we captured thermodynamic information, experimental conditions, and literature citations. We also standardized measurements and calculations across the data entries, including temperature in Kelvin, energy in kcal/mol, and Gibbs free energy (ΔΔ*G*) as in the formula:

$$\begin{equation*}\Delta \Delta G = \Delta G \left( {{\rm wild}{\hbox{-}}{\rm type}} \right) - \Delta G \left( {{\rm mutant}} \right)\end{equation*}$$

where negative ΔΔ*G* values indicate that the mutation has destabilized the protein and positive ΔΔ*G* values that the mutant protein is more stable.

On the first data acquisition stage, all 1,902 references in ProTherm were manually checked and validated. References that did not contain data about missense mutations were removed, leaving 829 papers. During this process, errors in data fields were corrected, duplicate entries were removed, and 329 new data-points not previously captured, but present in the original papers, were included.

New data were identified through manual literature curation. Optimized search terms (Supplementary Figure S1) were used to identify an initial pool of over 34,000 manuscripts available on PubMed. These were further narrowed down to those that contained experimental thermodynamic results for missense mutations. In total, 393 papers were analyzed and 5,654 new data points obtained, which were confirmed by at least two independent curators. Supplementary Figure S2 shows the distribution of unique mutations collected per year.

### Mutation annotation

Collected mutations were mapped to protein structures available at the Protein Data Bank using (31). Different characteristics of the wild-type residue environment were calculated, including secondary structure, torsional angles, relative solvent accessibility (32) and residue depth (33). Additional residue-level information used to annotate the mutations included different substitution matrix scores. Mutation annotations were calculated using the Biopython (34). Mutation effects are also depicted via pharmacophore modeling (23). Pharmacophore modeling has been introduced in the context of mutation analysis in a previous work (23) to characterise the effect of mutations based on the differences in atom counts per pharmacophore type. Mutations that do not map to any available experimental structures are still listed but without any structure-based features calculated.

### Database and web interface implementation

The database architecture was developed using SQLAlchemy, a database toolkit for Python (version 2.7). All data is stored in an SQLite database and available to download at http://biosig.unimelb.edu.au/thermomutdb/downloads. The backend system was developed using the Flask Python module (version 1.0.2) and the REStful API uses RestX extension for Flask (version 0.2.0). The web interface was implemented using the Bootstrap (version 4) framework. It also uses HTML5, CSS, JavaScript, and JQuery. JINJA2 templating language for Python was used to dynamically generate HTML templates.

## RESULTS

### Web interface and usage

ThermoMutDB contains information of the protein, mutational information, experimental methods and conditions, thermodynamic parameters, derived data, and literature information (details are available in Supplementary Table S1 and Figure S2). The database provides a user-friendly web interface that contains five modules: *Explore and Browse*, *Contribute*, *Downloads*, *API* and a detailed tutorial.

#### Explore and browse

**Figure 2.**
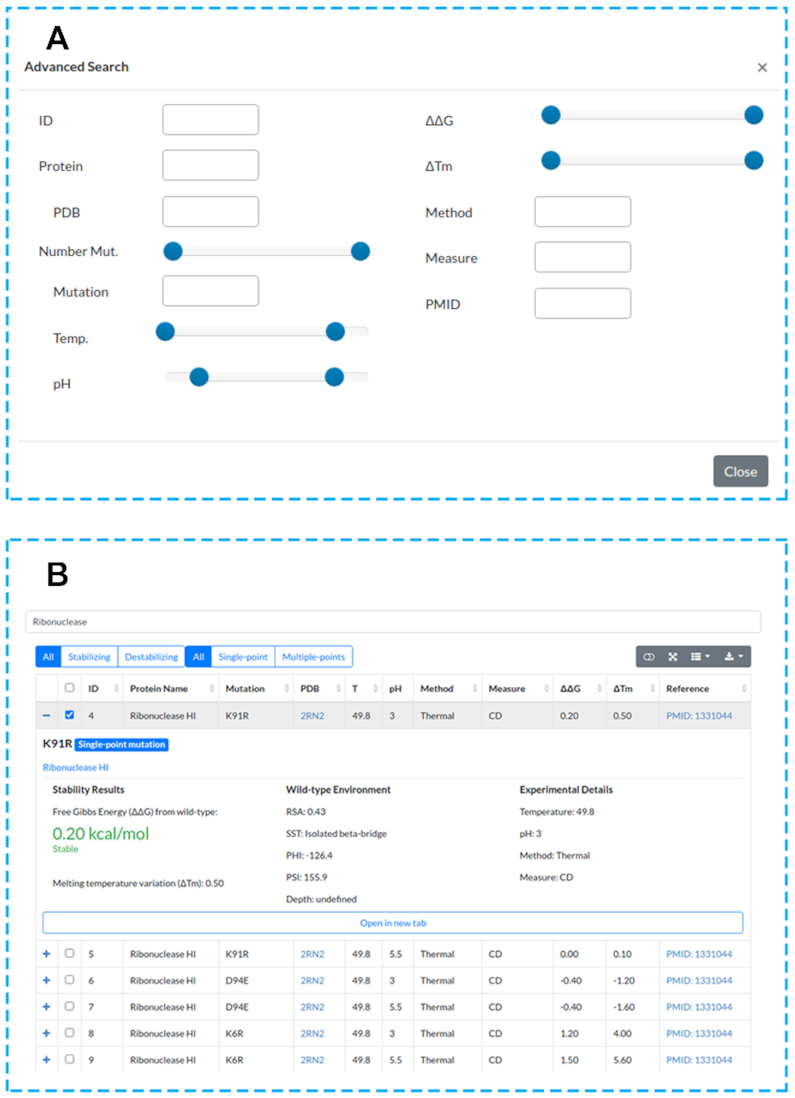
ThermoMutDB web interface search and results pages. (**A**) ThermoMutDB offers 12 query modes, with detailed information available about each query type through the ‘Help’ page at the top navigation bar and through on-page help in the form of question mark tooltips. (**B**) The general layout of the result page, showing a summary of information for each entry as well as detailed view.

In order to access the data, a search can be performed. This can be done either by selecting the ‘Browse’ page from the navigation bar or by writing the desired words on search input available on the ‘Home’ page. In both cases, users can use different filter combinations (Figure 2A), include or exclude columns, and download selected results in several formats (JSON, XML, CSV, TXT, SQL, MS-Excel and PDF).

The search results are shown in an interactive table, with columns providing experimental information recovered from literature and also derived properties (Figure 2B). Aiming to improve user experience, it is possible to visualize a summary for each entry by clicking on the ‘+’ icon. This option can lead to a ‘Details’ page that shows all information about the mutation and provides related files to download (Supplementary Figure S3).

#### User contributions

To facilitate a continuous database update, we have implemented a user's contribution section (Supplementary Figure S4), which allows the scientific community to share new data or identify potential errors that will be manually checked by our team. To submit contributions it is just required to fill the form with mutation and thermodynamics data, to inform a contact email and a reference (paper published, accepted, or pre-print). Although significant effort has been devoted to ensure high quality data curation, users have the option to report any issues with the data to our team. These are important efforts to further expand and improve the database.

#### Downloads

All data in the database can be downloaded from the ‘Download’ page in CSV or JSON formats. It is also possible to download the protein structure files related to data available.

#### Programmatic access via an API

ThermoMutDB supports programmatic access via a RESTful API to allow other services to harness our data easily. The ‘API’ page provides documentation of all endpoints available and allows users to execute queries using provided fields. Other queries can be performed by passing parameters through the URL (Supplementary Figure S5).

### Data statistics

**Figure 3.**
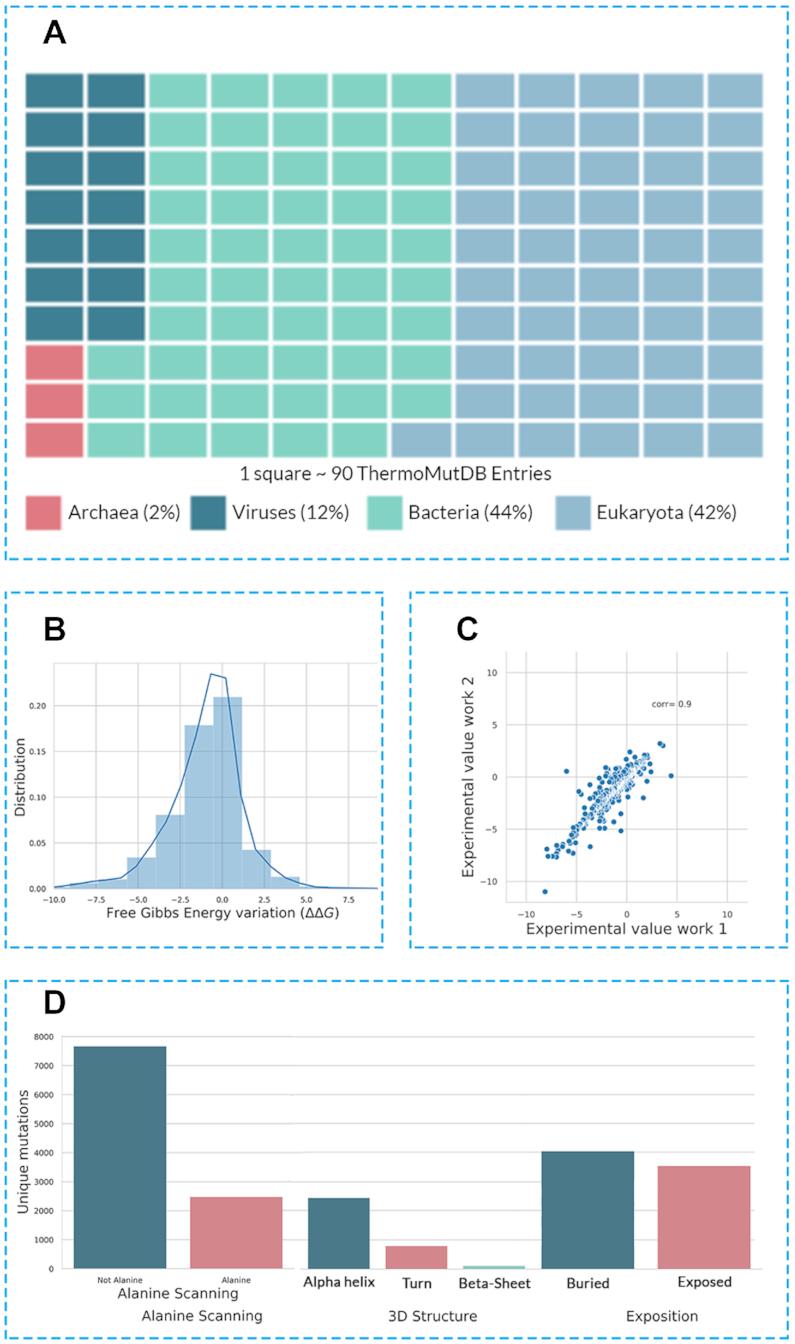
Composition of ThermoMutDB entries. (**A**) depicts the distribution of phylogenetic kingdoms of proteins in the database. (**B**) highlights the distribution of thermodynamic effects of mutation in the database, given as the variation in Gibbs Free Energy (ΔΔ*G*). (**C**) Experimental variability of mutation assessed under different conditions and groups. (**D**) Distribution of mutations in ThermoMutDB based on type (mutation to alanine/non-alanine), their location and residue environment.

Examining the distribution of mutations in the ThermoMutDB reveals a number of natural biases that need to be taken into consideration when developing, or evaluating, new predictive tools. ThermoMutDB contains thermodynamic information on 14,669 mutations across 588 proteins. This represents a significant increase over ProTherm, with a 83% increase in unique mutations and over 300 new proteins. Supplementary Figure S6 shows the distribution of unique mutations collected per year. The majority of these are single-point mutations (82.8%), with mutations to alanine being over-represented (Figure 3D). This becomes evident when we look at the distribution of wild-type and mutant amino acid residues within the database (Supplementary Figure S7). The most frequent mutations were from Leucine and Valine to Alanine, while 10 mutations were not present in the dataset, including W→G, W→P and C→K among others, which seem to denote large changes in residue physicochemical properties.

As would be expected by chance, two thirds of mutations within the database are destabilising (Supplementary Figure S8). This natural bias creates an extra challenge for computational methods built using this information, in particular those based on machine learning approaches, regarding the prediction of stabilising mutations, which are less well represented. It is important to note, however, that the data on ThermoMutDB represents an increase of over 100% in stabilising mutations in comparison with previous resources. No apparent correlation was identified between the mutation effects and their location within protein structures, with mutations leading to increased and decreased stability similarly distributed across protein structures when looking at residue depth (Supplementary Figure S9). Mutations in ThermoMutDB are spread across different protein classes (Supplementary Figure S10) and diverse in terms of secondary structure (Supplementary Figure S11).

Within ThermoMutDB, we identified mutations that had been experimentally measured at least twice and, by comparing the variance between these replicate results (Figure 3C), we identified a Pearson's correlation of 0.9. This provides a measure of the intrinsic noise in the data, and suggests a theoretical maximum performance that should be expected for predictive stability tools built using this data.

## DISCUSSION

ThermoMutDB represents a significant increase in availability, reliability and diversity of thermodynamics data linking effects of mutations to protein stability. We believe this resource will have a significant impact on understanding the effects of mutations on protein structure and stability. It will enable experimental scientists to identify previously characterised mutations in proteins of interest, and provide computational scientists with a comprehensive and refined set of experimental data to query the relationship between changes in protein sequence and stability, facilitating the development of new computational tools to analyse these relationships and develop prediction algorithms.

New mutation thermodynamics data collected and compiled in ThermoMutDB will also allow for more robust, comprehensive and independent validation of currently available computational predictors. The database will be continuously maintained and updated, enabling submission of user contributions and data access through an intuitive web-based interface (http://biosig.unimelb.edu.au/thermomutdb) as well as programmatic access through an API.

## Supplementary Material

gkaa925_Supplemental_File
