# Supplementary material for: ThermoMutDB: a thermodynamic database for missense mutations
Source: Nucleic Acids Res. 2020 Oct 23;49(D1):D475–9. doi: 10.1093/nar/gkaa925 (PMC7778973; doi:10.1093/nar/gkaa925)
Supplement: gkaa925_Supplemental_File [file gkaa925_supplemental_file.pdf]

ThermoMutDB: a thermodynamic database for missense mutations

Joicymara S. Xavier[1,2], Thanh-Binh Nguyen[3], Malancha Karmarkar[3,4],Stephanie Portelli [3,4], Pâmela M. Rezende[2], João P. L. Velloso[2], David B. Ascher[3,4,5*], Douglas E. V. Pires[3,4,6*]

[1]Institute of Agricultural Sciences, Universidade Federal dos Vales do Jequitinhonha e Mucuri;
[2]Instituto René Rachou, Fundação Oswaldo Cruz;
[3]Computational Biology and Clinical Informatics, Baker Heart and Diabetes Institute;
[4]Bio 21 Institute, University of Melbourne;
[5]Department of Biochemistry, University of Cambridge;
[6]School of Computing and Information Systems, University of Melbourne

*To whom correspondence should be addressed. Tel: +61 3 8344 8185; Email: douglas.pires@unimelb.edu.au. Correspondence may also be addressed to David B. Ascher. Email: david.ascher@unimelb.edu.au.

protein AND (mutant* OR mutat* OR variant*) AND ((destabili* OR stabili* OR thermostab*) OR (kcal/mol OR kj/mol OR kcal mol OR kj mol) OR (“free energy” OR “gibb* free energy" OR "melting temperature")) NOT(review[Publication Type] OR "molecular dynamics" OR predict*)

Figure S1: Search query used to identify and collect mutation thermodynamics data from publications available on PubMed. This query was designed to encompass mutations in proteins and their experimentally measured effects while excluding works involving their silico characterization.

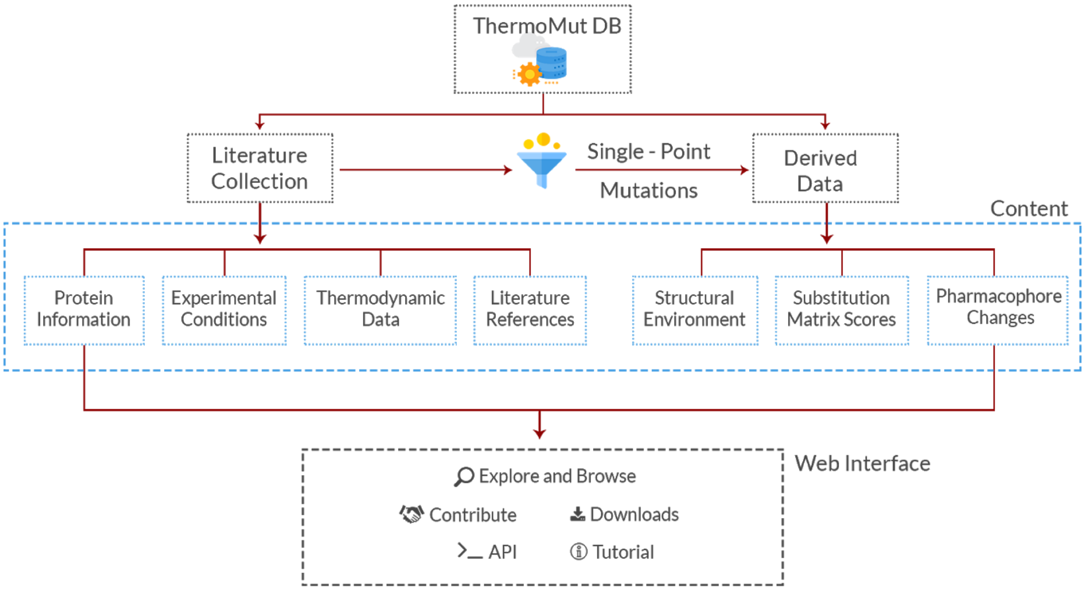

Figure S2: Schematic workflow of ThermoMutDB data organization.

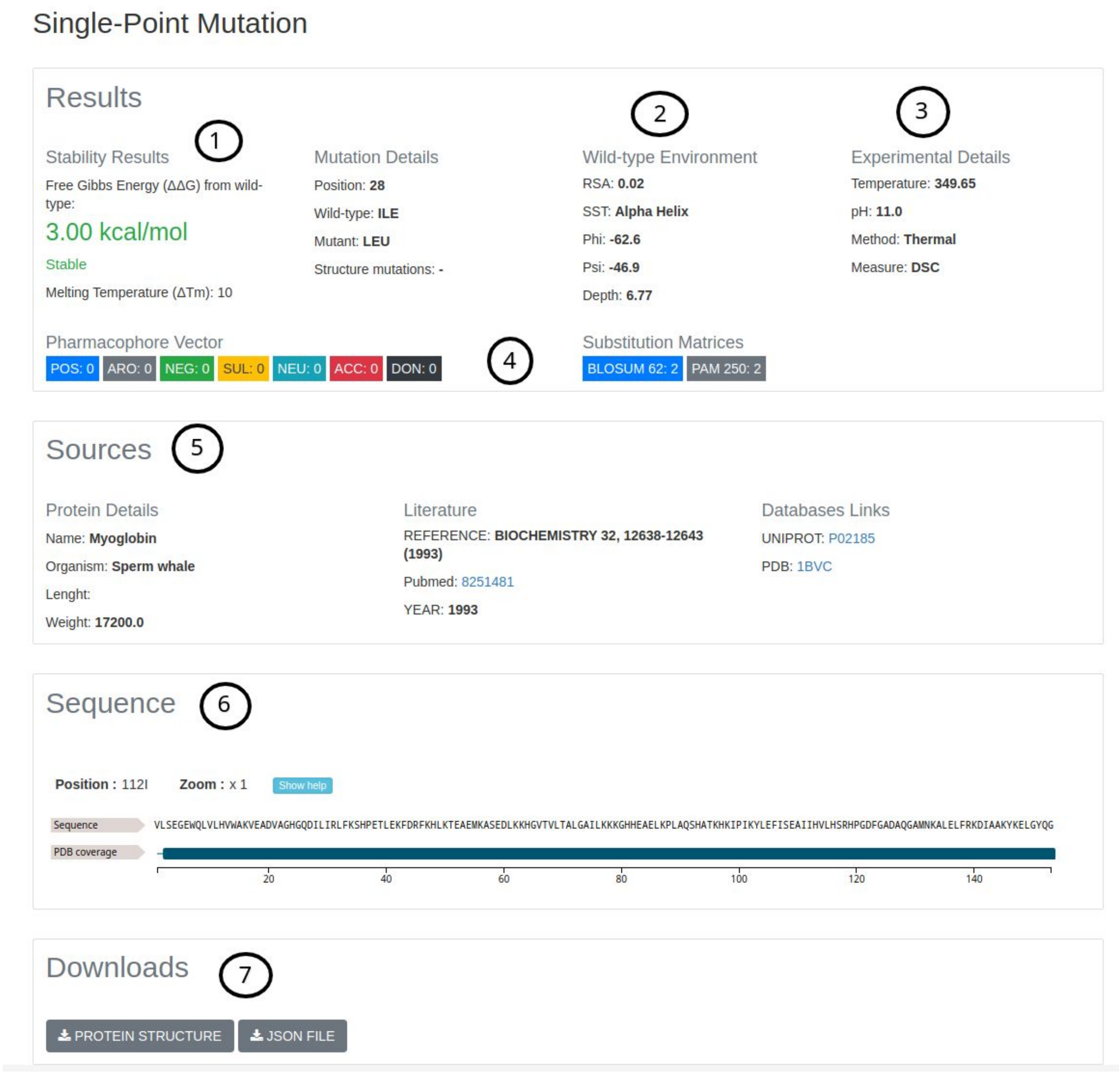

Figure S3: ThermoMutDB mutation details page. The mutation page provides information about the mutation and its thermodynamic effects (1), residue environment properties (2), experimental conditions (3), as well as substitution matrix scores for the mutation and their effects in terms of pharmacophore changes (4). In this page, the literature information is provided, with links to external databases (5), followed by the alignment of PDB sequence against the Uniprot sequence (6), as well as the option to download the entry and protein structure (7).

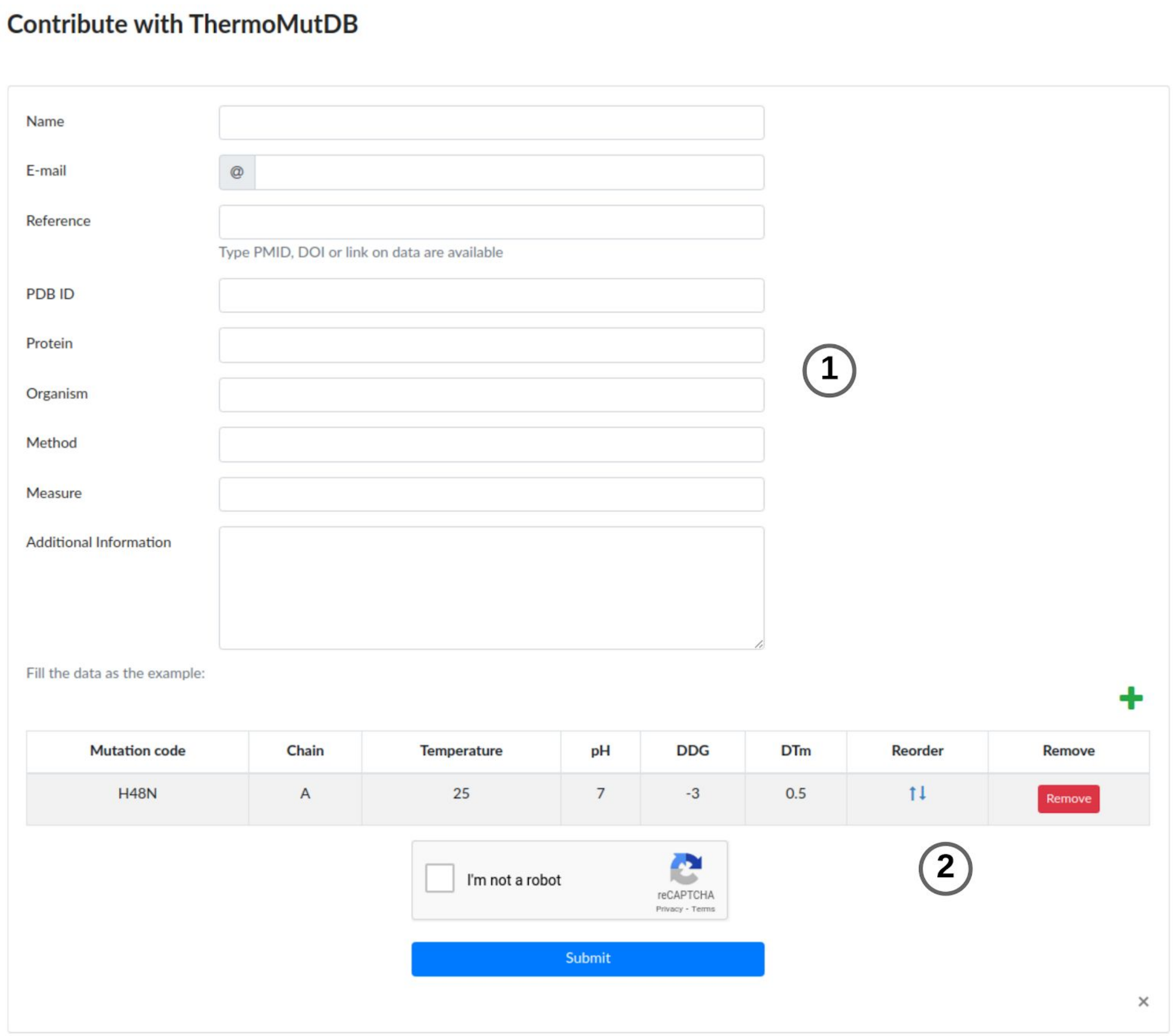

Figure S4: User contribution page. ThermoMutDB allows users to submit their own contributions to be manually curated and incorporated into the database. Users are requested to provide information on the publication, protein and experimental protocol (1) and can include thermodynamic information for one or more mutations (2).

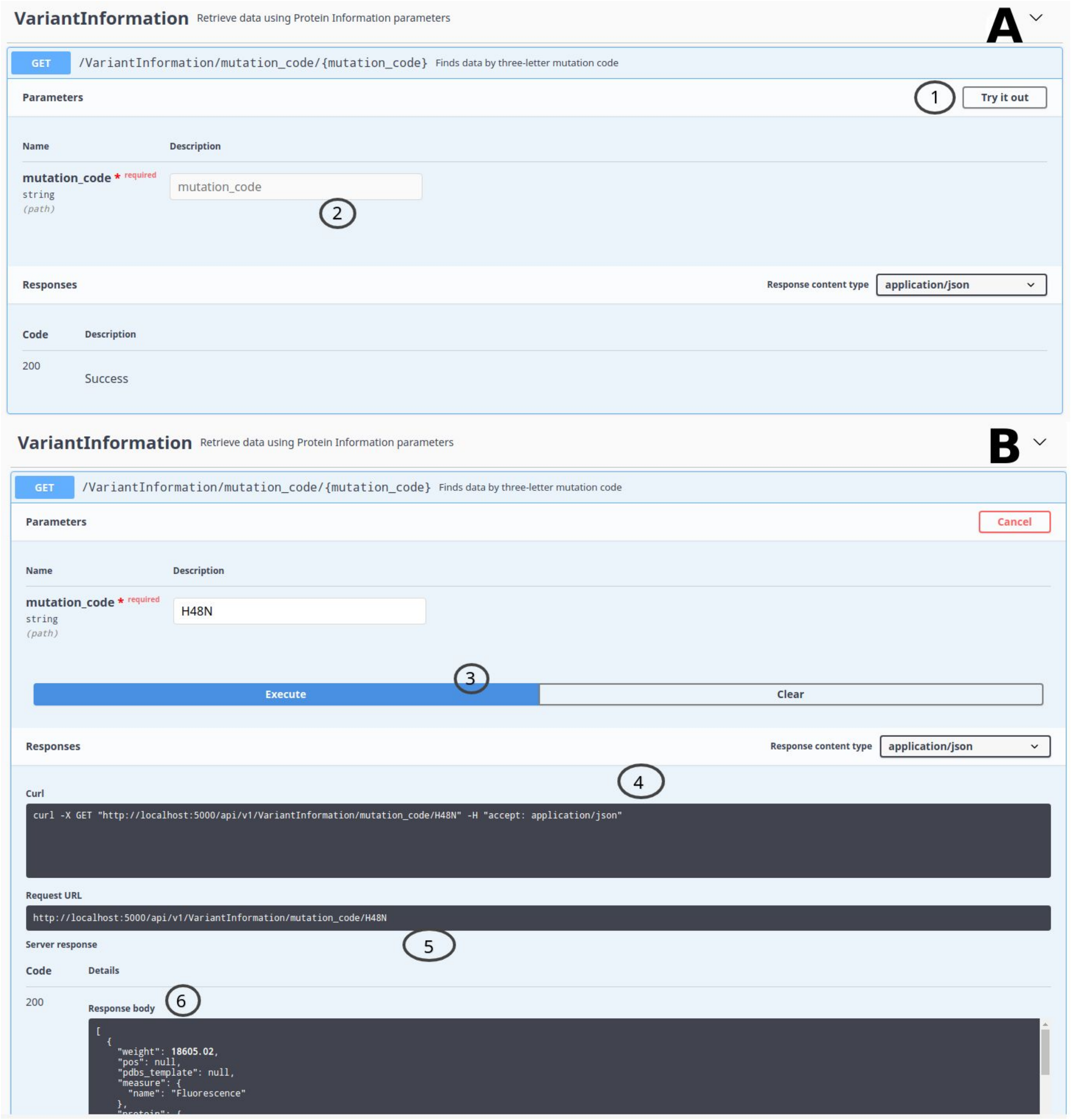

Figure S5: Programmatic Access Via an API. The figure depicts advanced search options for ThermoMutDB and respective URL parameters. (1) To start, click on “Try it out”, (2) type the desired parameters, and (3) click on “Execute” button. The response shows (4) Curl command, (5) URL request, and (6) the response body.

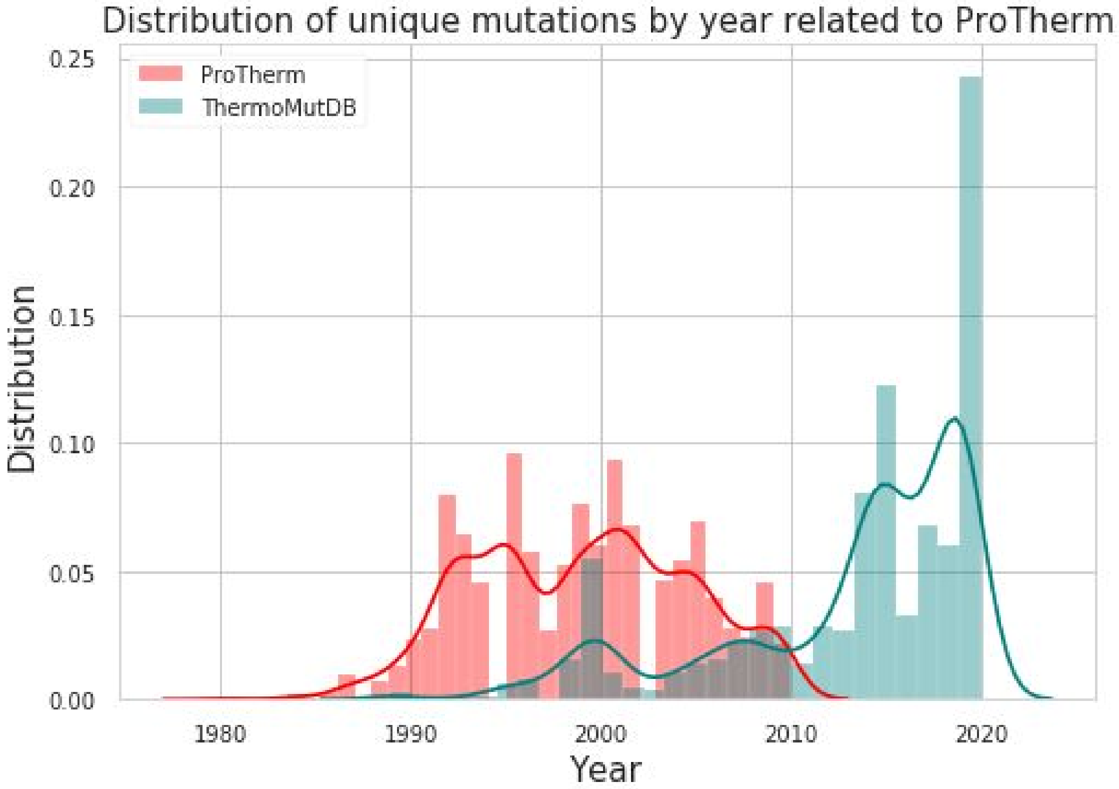

Figure S6: Distribution of entries on ThermoMutDB over the years, highlighted by origin.

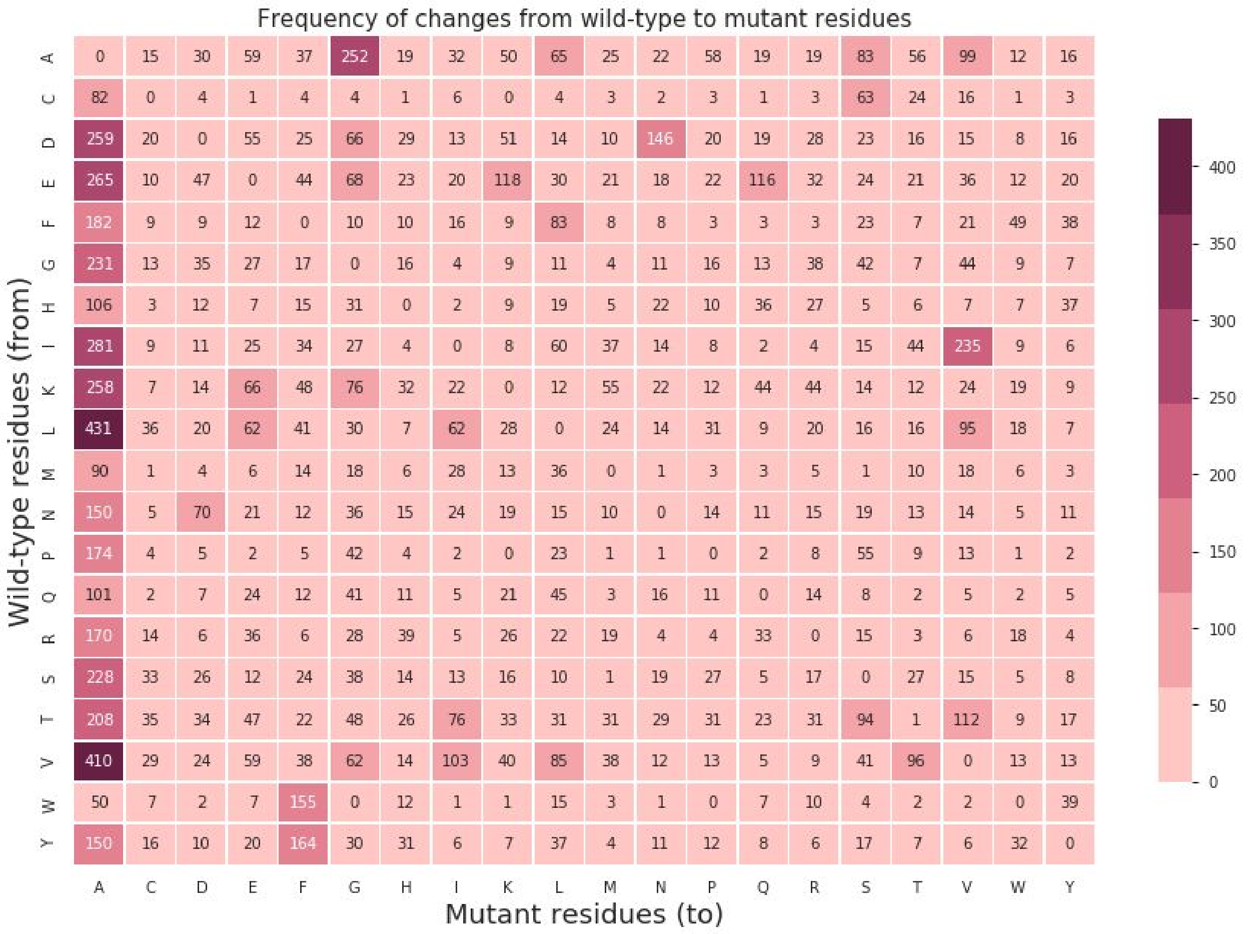

Figure S7: Frequency of changes from wild-type to mutant residues within the ThermoMutDB. The majority of mutations are to alanine, characterising alanine-scanning experiments.

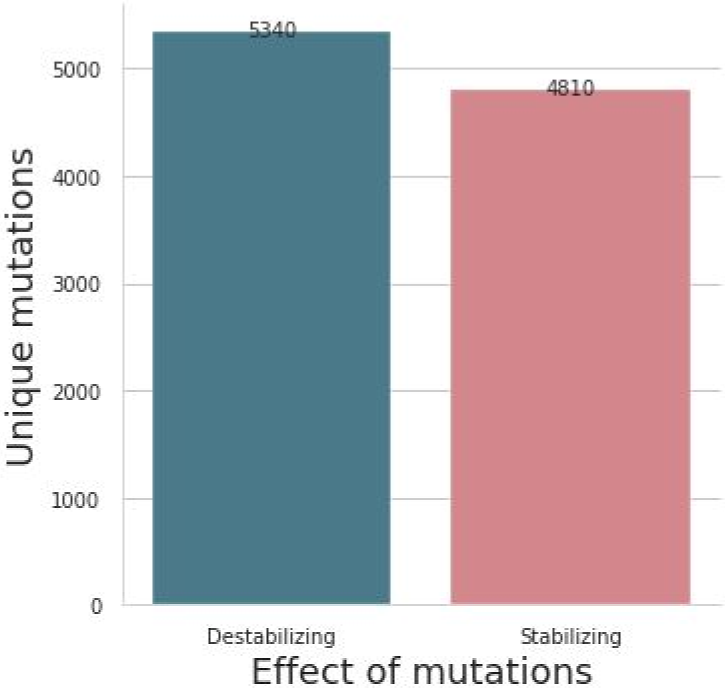

Figure S8: ThermoMutDB entries divided based on mutation effects.

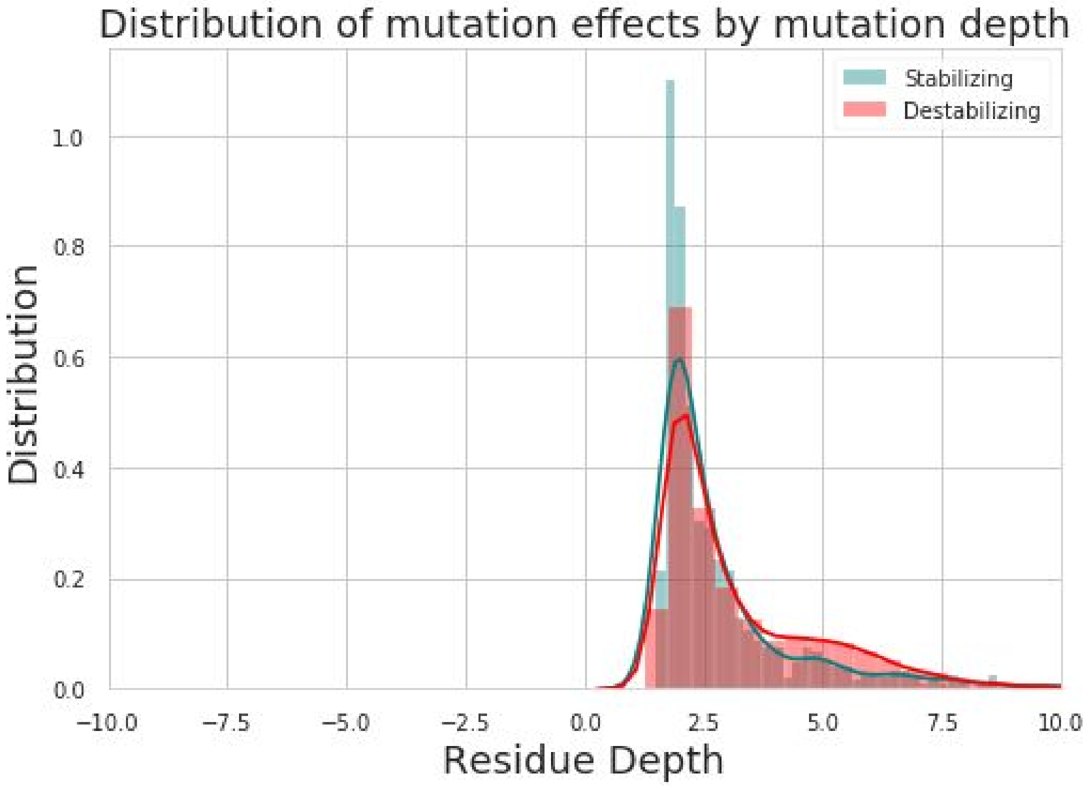

Figure S9: Distribution of residue depth for stabilising and destabilising mutations in ThermoMutDB.

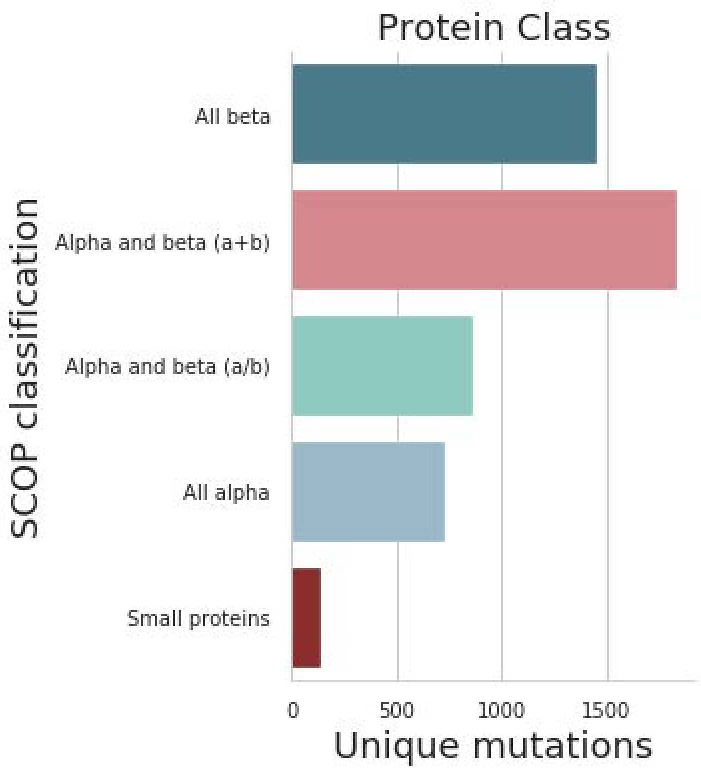

Figure S10: Distribution of mutation on ThermoMutDB based on protein classification according to SCOP.

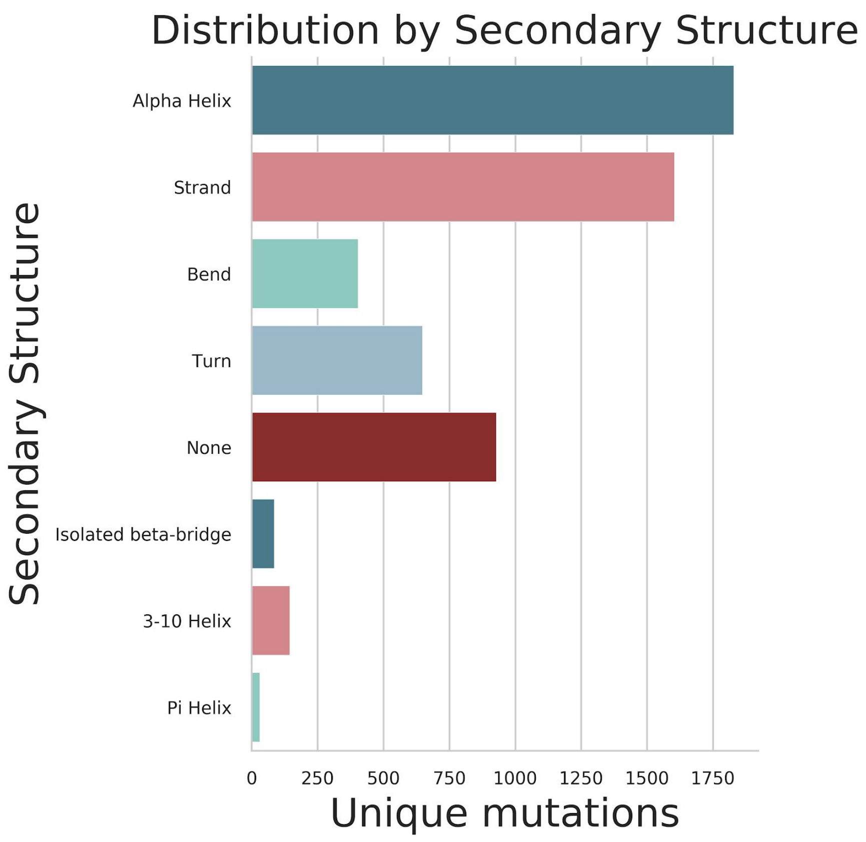

Figure S11: Distribution of mutation on ThermoMutDB based on secondary structure.

Table S1: Information content of ThermoMutDB

Content	Description	Measure (if there is)	single	multiple
**Protein Information**				
Protein name	Protein Name		X	X
Source	Protein Organism		X	X
Uniprot	Uniprot Code		X	X
PDB wild	Protein Data Bank code		X	X
PDB mutant	PDB code for mutant (when is available)		X	X
PDBs template	PDBs used as a template to model wild_type (when is available and necessary)		X	X
Length	Length of sequence		X	X
Weight	Molecular weight		X	X
PIR ID	Protein Information Resource		X	X
SWISSPROT ID	Code of Swiss-Prot (Revised Entries)		X	X
Mutation	Three-digits mutation code		X	X
Mutated chain	Mutated chain		X	X
Structure mutations	Number of mutations in the structure		X	X
Structure coverage	Structure coverage		X	X
Mutation count	Number of mutations		X	X
**Experimental Conditions**				
Temperature	Experimental temperature	Kelvin (K)	X	X
pH	Experimental pH		X	X
Measure	Experimental techniques for studying protein folding.		X	X
Method	Techniques to denature a protein		X	X

**Thermodynamic data**				
ΔΔG	Variation of Free Gibbs Energy on the experiment	$kcal/mol^{-1}$	X	X
ΔTm	Variation of Melting Temperature on the experiment	Kelvin (K)	X	X
**Structural environment**				
SST	Secondary Structure classification		X	
RSA	Relative accessible surface area		X	
PHI	Phi angle value		X	
PSI	Psi angle value		X	
Residue Depth	The average distance of atoms of wild-type residue from the solvent accessible area		X	
CA Depth	The average distance of atoms of CA from the solvent accessible area		X	
Relative B Factor	Temperature factor		X	
**Substitution matrices scores**				
Blosum 62	BLOSUM 62 matrix score		X	
Pam 250	PAM 250 matrix score		X	
**Pharmacophore changes**				
POS	Positive		X	
NEG	Negative		X	
ACC	Hydrogen bond acceptors		X	
DON	Hydrogen bond donors		X	
ARO	Aromatic rings		X	
SUL	Sulfuric acid		X	
NEU	Neutral		X	
**Literature information**				
Reference	Publication reference		X	X
PMID	Pubmed code of publication		X	X

DOI	Digital Object Identifier of publication		X	X
YEAR	Year of publication		X	X
